# Radiological analysis and outcomes of isolated greater tuberosity fracture-dislocations

**DOI:** 10.1308/rcsann.2023.0019

**Published:** 2023-08-23

**Authors:** J Saleem, B Guevel, E Gillott, C Mitchell, A Widjono, A Qavi, P Domos

**Affiliations:** ^1^ Royal Free NHS Foundation Trust, UK; ^2^ Imperial College London, UK

**Keywords:** Humerus, Fracture, Dislocation, Greater tuberosity, Shoulder

## Abstract

**Background:**

The purpose of this study was to investigate different radiological characteristics for isolated greater tuberosity (GT) fracture-dislocations and their effects on complication and reoperation rates.

**Methods:**

A two-centre, retrospective study was performed on patients with a minimum 1-year follow-up (median 4.5 years). Patients were split into two groups, Group A (<65 years old) and Group B (≥65 years old). Outcomes included initial injury characteristics (dislocation and fracture type, AC/BC ratio and distances), the reduction environment and postreduction outcomes including complications.

**Results:**

A total of 55 patients were included in this study, with a reduction in the emergency department (ED) performed in 93% of patients. Complication rates (47% overall) were similar in both groups, with an overall nonunion rate of 27%. No nonunions occurred in fractures reduced in theatre compared with 29% occurring in reductions in ED (*p*<0.001); 11% of patients experienced surgical neck fractures, the majority of which were in Group B (*p*=0.003). A larger fracture fragment (i.e. higher AC/BC or AC distances) was correlated with a higher incidence of nonunion in Group B compared with Group A (*p*=0.003), and a higher risk of stiffness in both groups (*p*=0.049); 16% of patients demonstrated delayed displacement of their GT.

**Conclusions:**

This study highlights the high complication rates associated with these injuries. Age and specific radiological parameters should be taken into consideration when risk stratifying, as should reducing these fractures in a theatre setting. Interval radiographs are also advised to monitor GT displacement for at least 2–3 weeks.

## Introduction

Proximal humeral fractures are common, especially in the osteoporotic patient, and isolated fractures of the greater tuberosity (GT) of the shoulder account for 14–20% of proximal humeral fractures.^1–4^ Traumatic dislocations of the shoulder have a reported incidence of 17–23.9/100,000 and are associated with GT fractures in 15–25% of cases.^5–7^ In these circumstances, the fragment is dorsally displaced by the attached rotator cuff, and it has been suggested that the degree of residual displacement after reduction can be prognostic of restoration of shoulder function long term.^8,9^ Operative management of a GT fragment that is displaced more than 3–5mm has been suggested.^10–12^ Furthermore, studies suggest that two-thirds of these fractures were initially missed at primary evaluation due to osseous overlap, which causes difficulties in their identification.^13^ Importantly, a malunited GT can cause impingement and rotator cuff dysfunction, ultimately leading to impaired shoulder function.^14,15^

Previous studies have evaluated the role of different morphological classifications and radiological characteristics in evaluating and prognosticating these injuries. Mutch *et al* described a morphological classification for GT fractures, and concluded that certain fracture subtypes would likely have implications on their respective management.^16^ Guo *et al* found a statistically significant relationship between the size of GT fragment in fracture-dislocations and the risk of iatrogenic humeral neck fractures during reduction.^17^ Furthermore, a paper by Saupe *et al* evaluated the role of the “acromio-humeral distance” and its statistically significant role in determining rotator cuff injuries, although this was not investigated in the fracture-dislocation population.^18^

GT fracture-dislocations can lead to long-term complications in patients, and the role of reduction management and radiological parameters on outcomes identified in these papers remains unclear. Furthermore, the impact of these injuries in the young versus the elderly also remains unclear. The purpose of this study was to investigate the role of GT fracture-dislocation clinical and radiological characteristics, their management and their associated outcomes and complications.

## Methods

### Study design

A two-centre, retrospective analysis was performed of the radiological and clinical outcomes of patients who sustained an isolated GT fracture-dislocation between January 2012 and February 2020. Patients included all adult patients (≥18 years old) with a minimum 12-month follow-up. The exclusion criteria were patients with any other fracture type. Patients were stratified into those below age 65 years (Group A) and those aged 65 years or above (Group B).

Patient records were analysed by two independent reviewers to collect the following data.

### Initial injury characteristics

**Figure 1:**
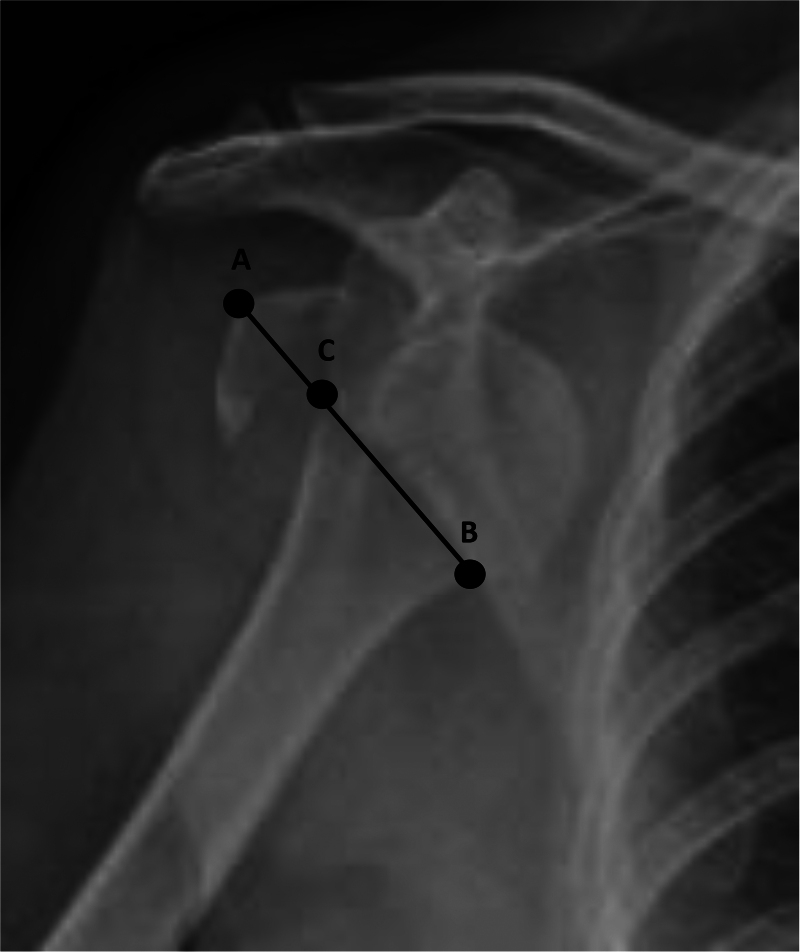
Illustration of points A, B and C. Permission to reproduce image obtained from Guo *et al.*

This included dislocation and fracture types, comminution and radiological measurement characteristics. In regard to fracture types, this was determined as per the paper by Mutch *et al*.^16^ This details avulsion fractures as a GT fragment with a horizontal fracture line, a split fracture as having a vertical fracture line and a depressed fracture as a fragment displaced inferiorly onto the humeral head. Regarding radiological measurement characteristics, the “AC”, “BC” distances and the “AC/BC” ratio were determined as described by Guo *et al* as this is one of the few papers to have characterised injuries of this nature.^17^ On an anterior-posterior view of the shoulder point “A” is the vertex of the fractured GT, point “B” is the maximum curvature of the medial cortex between the humeral surgical neck and anatomical neck and point “C” is at the fracture line where it crosses “AB”. The AC distance in effect denotes the degree of fracture fragment size/displacement. This is illustrated in Figure 1.^17^

### Shoulder reduction method

The location of reduction in either the emergency department (ED) or operating theatre.

### Primary (initial) post-injury outcomes

These data comprised initial and delayed displacement (>5mm), the timing of delayed displacement and iatrogenic surgical neck fractures resulting from reduction.

### Secondary (late) post-injury outcomes

These data included postinjury complications such as nerve injury or stiffness, operations performed postinjury and final radiological union outcomes. The latter included union, malunion (defined as >5mm displacement), delayed union (>6 weeks) and nonunion (>6 months). Nerve injuries or stiffness were identified using available clinical documentation.

### Data analysis

Patient outcomes with continuous variables were presented as a median with an interquartile range (IQR). Other outcomes were reported alongside the overall percentage.

Statistical analyses were performed using Microsoft Excel. Unpaired, two-tailed *t*-tests were used to assess for statistical significance in continuous data and Pearson’s chi-square test for categorical data. A regression analysis was performed to ascertain relationships between fracture-dislocation variables and outcomes. Pearson’s correlation was used to look for correlation between age and radiographic findings. A point biserial correlation was used to look for correlation between age and outcomes and radiographic findings and outcomes. The Evans scale for reporting strength of correlations was used to determine the strength of these correlations.^19^ A chi-square test was used to look for statistically significant differences in complications for types of dislocations, comparing complications with expected complications for each age group.

## Results

**Figure 2:**
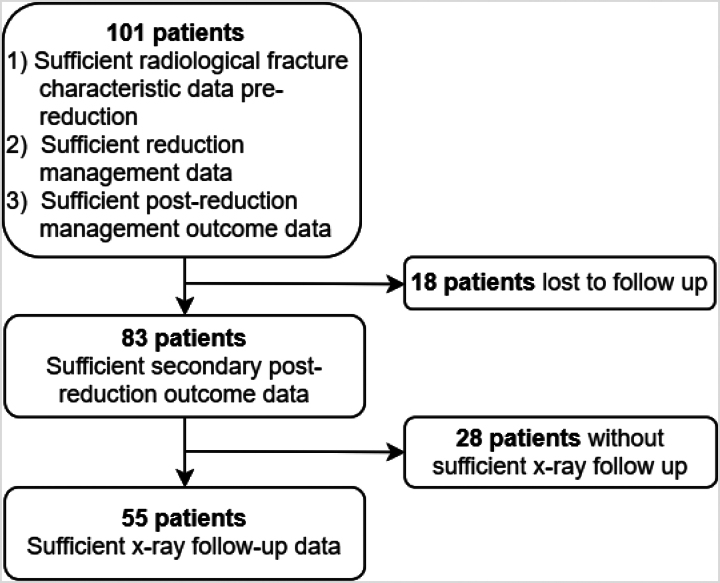
Flowchart demonstrating patient study pathway

Fifty-five patients were included with a median follow-up time of 4.5 (2.5–6.3) years. Figure 2 demonstrates the patient follow-up pathway.

The median age at presentation was 66 (57–74) years old; 25 (45%) patients were stratified into Group A, and 30 (55%) into Group B. A total of 20 patients (44%) were male, and there were significantly more male patients in Group A (16) as compared with Group B (8) (*p*<0.05). Subcoracoid fracture-dislocations (47) represented 85% of all presentations. Avulsion fractures (60%) were the commonest fracture type with 33 patients sustaining this type of injury; 36 (65%) fractures had some degree of comminution. There were no statistically significant differences between Groups A and B regarding laterality, the type of dislocation, the fracture type or the presence of comminution (Table 1).

**Table 1:** Patient characteristics

	All patients	Group A	Group B	*p* value
*N*	55	25	30	
Sex (*n*)				
Male	24	16	8	**0**.**005**
Female	31	9	22	**0**.**005**
Age (years)				
Median (IQR)	66 (56.5–73.5)	55 (36–59)	73 (68.25–81.75)	**<0**.**001**
Shoulder laterality (*n*)				
Right	30	17	13	0.067
Type of dislocation (*n*)				
Subcoracoid	47	20	27	0.294
Subglenoid	1	1	0	0.895
Subclavicular	7	4	3	0.506
GT fracture type (*n*)				
Avulsion	33	14	19	0.580
Split	20	11	9	0.283
Depression	2	0	2	0.665
Comminution (*n*)				
Yes	36	15	21	0.437
No	19	10	9	0.447

GT = greater tuberosity; IQR = interquartile range.

Regarding overall outcomes, 51 (93%) patients had a reduction performed in ED whereas 4 (7%) patients had a reduction in theatre (Table 2); 26 (47%) patients had an undisplaced GT fracture postreduction, thereby 29 (53%) were displaced. Of those 26 patients with an undisplaced fracture postreduction, 9 (45%) thereafter demonstrated delayed displacement, the diagnosis of which was made a median of 12 (8.0–17.0) days postinjury. Postreduction, six (11%) of all fractures resulted in a surgical neck fracture. Of the 55 patients with follow-up data, complications occurred in 26 (47%) patients comprising 10 with nerve injury (18%), 14 with stiffness (25%) and 2 with redislocation (4%) (Table 3). Eight patients (15%) required a manipulation under anaesthesia (MUA) after a failed ED reduction, and there was a total of 20 further operations following a successful reduction, of which there were 15 open reduction and internal fixation (ORIFs). The ORIFs included three plate fixations, three screw fixations, seven anchor fixations and two transosseous suture fixations. Furthermore, there was one hemiarthroplasty and one reverse total shoulder replacement. Three patients had more than one operation. Regarding union outcome follow-up data, 40 patients (73%) achieved overall union; however, 25 (63%) of these patients demonstrated delayed union. The overall nonunion rate was 27%, comprising 15 patients.

**Table 2:** Fracture dislocation characteristics

	All patients	Group A	Group B	*p* value
*N*	55	25	30	
Prereduction characteristics				
AC distance (mm)				
Median (IQR)	20.4 (15.4–23.9)	19.8 (12.9–23)	21 (16.4–25.4)	0.067
BC distance (mm)				
Median (IQR)	41.3 (38–47.4)	44.6 (39.5–49.6)	39.7 (35.6–44.1)	0.**040**
AC/BC ratio				
Median (IQR)	0.48 (0.34–0.63)	0.40 (0.29–0.59)	0.55 (0.38–0.66)	0.**019**
AH (mm)				
Median (IQR)	71.3 (65.8–77.9)	72.5 (65.6–78.3)	69.8 (66.1–74.8)	0.240
Postreduction characteristics				
Reduction location				
Emergency department				
*N* (%)	51 (93)	23 (92)	28 (93)	0.788
Theatre				
*N* (%)	4 (7)	2 (8)	2 (7)	0.789
Un-displaced				
*N* (%)	26 (47)	12 (48)	14 (47)	0.887
Surgical neck fractures				
*N* (%)	6 (11)	1 (4)	5 (17)	**0**.**003**
Displaced immediately postreduction				
*N* (%)	29 (53)	13 (52)	16 (53)	0.887
Displacement delayed postreduction				
*N* (%)	9 (16)	6 (24)	3 (10)	**0**.**008**
Days to delayed displacement				
Median (IQR)	12.0 (8.0–17.0)	15.0 (12.3–20.1)	8.0 (7.5–10.0)	0.079
Postreduction displacement AC (mm)				
Median (IQR)	9.0 (7.15–11)	8.1 (6.9–11.1)	9.0 (7.9–10.2)	0.181

Prereduction fracture characteristics were similar across both groups; however, the younger patient cohort demonstrated a lower AC/BC ratio (0.40 versus 0.55) (Table 2). There were six surgical neck fractures postreduction, five of which occurred in Group B, with this difference between subgroups achieving statistical significance (*p*=0.003). The time to delayed displacement was longer in Group A (15 days) as compared with Group B (8 days), and Group A were more likely to demonstrate delayed displacement (*p*=0.008).

Avulsion fracture types demonstrated a high frequency of nonunions across both Groups, with a higher occurrence in Group B (42%) versus Group A (21%). Furthermore, across subgroups, there were no nonunions occurring in fractures reduced initially in theatre, as compared with the nonunion rate of 29% of fractures reduced initially in ED—a result that achieved statistical significance (*p*<0.001). Moreover, in those with a successful ED reduction across age groups, the nonunion rate was 8%, whereas the nonunion rate in those with an unsuccessful ED reduction was 45%, also significant (*p*=0.002).

Group A had a lower nonunion rate involving five patients (20%) as compared with ten patients in Group B (33%). Rates of stiffness (*p*=0.626), nerve injury (*p*=0.461) and total complications (*p*=0.395) were similar across Groups A and B. Regarding postinjury operations, a higher percentage of patients in Group B (43%) required operative intervention as compared with Group A (28%), and this achieved statistical significance (<0.001). Point biserial analysis showed some correlation between older age and the presence of comminution (0.203). Pearson correlation analysis showed weak negative correlation with increasing AC distance and GT nondisplacement postreduction (-0.284), as well as a weak positive correlation with increasing AC distance or AC/BC ratio and iatrogenic surgical neck fractures (0.286 and 0.263, respectively) needing an operation (0.263), and any complication (0.340). Furthermore, older age was weakly correlated with nonunion (0.315).

Regarding the relationship between radiological fracture-dislocation characteristics and outcomes, of the fifteen patients sustaining a nonunion, twelve patients (80%) had an increased AC distance as compared with three patients (20%) across age groups. Furthermore, patients with a higher BC distance, ten (67%), was associated with a higher frequency of union across age groups, as compared with those five (33%) with a lower distance. A higher AC/BC ratio was noted to have a significantly higher occurrence of nonunion in Group B (47%) as compared with Group A (13%) (*p*=0.003). With respect to complications, an increased AC/BC ratio was associated with a significantly increased risk of stiffness across age groups (69% versus 31%) (*p*=0.049), as well stiffness being significantly more common in Group B, eight patients, (50%) as compared with Group A, three patients (19%) (*p*<0.001). An increasing AC distance also increased the risk of nerve injury across age groups (nine patients (64%) versus five patients (35%)).

## Discussion

This study has provided a comprehensive overview of the outcomes for all isolated GT fracture-dislocations presenting to two trauma units across a period of 8 years.

Our demographic data were in keeping with similar studies.^20^ There was a bimodal distribution of the injury with a significantly higher proportion of younger male patients and older female patients, again in keeping with previous epidemiological studies.^21^ The median age of 66 years was also akin to similar studies, likely due to the reduced bone density at the proximal humerus in this age group.^16,22^ Mutch *et al* found a higher rate of split type fractures (41%), whereas in our study the majority were avulsion, potentially due to our older median age cohort having associated rotator cuff tears.^16^

The majority (93%) of patients had a reduction in ED and, if this was unsuccessful, there was a 45% chance of nonunion. Significantly there were no nonunions for fractures that were reduced first in theatre (*p*<0.001). One explanation for this could be the importance of attaining increased muscle relaxant to improve fracture reduction. In our paper, ED clinicians performed the ED reductions, as is common nationally. Furthermore, studies have demonstrated fracture-dislocations reduced in ED having a higher risk of further iatrogenic humeral fracture, which in turn could lead to a higher risk of nonunion as the humeral head becomes devascularised.^23,24^ They recommended these fractures be reduced under general anaesthesia and with enhanced muscle relaxation to mitigate this risk. It should be stated, however, that this inference is limited by the small number of patients in this paper who underwent their first reduction in theatre.^4^ This makes it difficult to draw conclusions in patient group sizes, which show a large disparity. In our study, 11% of patients, all but one of which were in the older age group, suffered from a postreduction humeral neck fracture. Additionally, 15% required an MUA after a failed ED reduction. Consideration therefore should be given to reducing these injuries in theatre, although other factors such as fracture type/size and displacement may also contribute.

Our study used a recently published ratio known as the AC/BC ratio to measure GT fracture size and displacement.^17^ Guo *et al* noted that a AC/BC ratio higher than 0.4 was significantly associated with a greater risk of iatrogenic humeral neck fracture (IHNF).^17^ This study found that higher AC/BC ratios were correlated with a higher risk of IHNF as per the paper by Guo *et al*, but in addition we noted it was also associated with a significant increase in stiffness across all age groups as well as nonunion, especially among the elderly. The increase in stiffness with larger GT fragments is theorised to be due to a combination of soft tissue adhesions/contractions, the anatomic constraints of the GT below the acromion and an alteration in rotator cuff biomechanics.^20,25^ Therefore, consideration of the AC/BC ratio should be taken when treating these fractures, as well as appropriate counselling on the increased risk of nonunion and the need for appropriate rehabilitation and physiotherapy.

**Figure 3:**
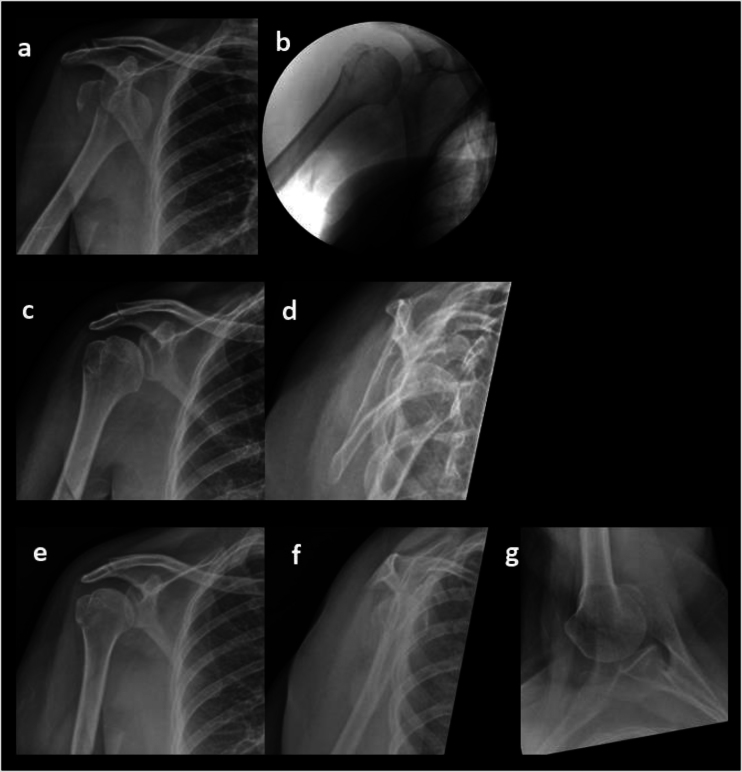
Delayed displacement following greater tuberosity fracture-dislocation. (a) AP radiographs of left shoulder with anterior GT fracture dislocation. (b) Intraoperative AP view following successful closed reduction with minimally displaced GT fragment; (c,d) AP and Y view radiographs showing no displacement of GT fragment at 1 week follow up. (e–g) AP, Y view and axillary view radiographs at 3 weeks showing displaced GT fragment. AP = anteroposterior; GT = greater tuberosity; Y = lateral scapula shoulder

The median time of 12 days to delayed displacement of a previously undisplaced fracture of the GT indicates the need for close follow-up in the first 3 weeks, as demonstrated in Figure 3. This is especially important in the younger age group who are more likely to have an intact rotator cuff, acting as a deforming force leading to a higher rate of delayed displacement. This was corroborated in this study where the younger age group were significantly more likely to suffer from delayed displacement. Regular follow-up for this cohort is also important due to the observed high complication rate. This comprised mainly nerve injury (18%) and stiffness (25%) with the redislocation rate being quite low at 4%, in keeping with a similar study by Dussing *et al.*^14^ The high rate of post-traumatic stiffness can paradoxically lead to reduced risk of instability due to the soft tissue contractures keeping the humerus in joint. Furthermore, the GT fracture likely reduces the joint compression force, reducing the chance of a labral tear, also reducing the redislocation risk. Operative intervention therefore should be targeted fixation of larger displaced fragments to reduce the risk of nonunion, rather than any stabilisation procedures.

The strength of our study was the comprehensive nature of the investigation of factors that could affect outcomes and guide management. To our knowledge, this is the first study to investigate the effect of this specific range of radiological parameters on outcomes.^17,26^ Radiographs are widely available, making this assessment accessible in low-income settings. Limitations of the study include its retrospective nature and being limited to two centres, thereby introducing an element of selection bias; however, all adult participants were included over an 8-year period. Furthermore, stiffness and nerve injury outcomes were identified through clinical documentation; therefore, no objective measurable criteria could be used to equate symptom severity between patients. Moreover, variables such as previous shoulder conditions and surgeries were not evaluated, which may be confounding factors. The displacement of a GT fragment is three-dimensional and therefore the use of radiographs instead of computer tomography (CT) scans is a limitation, although the use of CT scans to evaluate these injuries is not common. Larger studies across multiple sites with higher patient numbers are needed to investigate these associations further.

## Conclusion

To conclude, these injuries are associated with a high complication rate including delayed displacement, especially in younger patients, and surgical neck fractures. Our results suggest that the larger the fragment, the higher the risk of nonunion and stiffness, especially in the elderly. We would therefore recommend that patients with fracture-dislocations over the age of 65 years with an AC/BC ratio of greater than 0.4 are reduced in theatre to minimise potential complications. Patients should also be counselled on the high nonunion rate and the need for early physiotherapy to reduce the rate of post-traumatic stiffness.

In summary, although GT fracture-dislocations are classified as one general fracture type, our study has highlighted how different radiographic features associated with these injuries can have varying effects on outcomes. By publishing our data on how age, fracture configuration and initial management all have varying impacts on complication and union rates, we hope to guide a treatment algorithm for these injuries.

## Availability of data and material

The authors confirm that the data supporting the findings of this study are available within the article and/or its supplementary materials.

**Table 3:** Postreduction complication data

Fracture union outcomes				
	All patients	Group A	Group B	*p* value
*N*	55	25	30	
Union *n* (%)	15 (27)	9 (36)	6 (20)	0.439
Delayed union *n* (%)	25 (45)	11 (44)	14 (47)	0.549
Nonunion *n* (%)	15 (27)	5 (20)	10 (33)	0.197
**Clinical complications**				
	**All patients**	**Group A**	**Group B**	***p* value**
*N*	55	25	30	
Stiffness *n* (%)	14 (25)	6 (24)	8 (27)	0.626
Redislocation *n* (%)	2 (4)	1 (4)	1 (3)	0.700
Nerve injury *n* (%)	10 (18)	4 (16)	6 (20)	0.461
Total complication *n* (%)	26 (47)	11 (44)	15 (50)	0.395
Operations postreduction *n* (%)	20 (36)	7 (28)	13 (43)	**<0**.**001**
Reoperation *n* (%)	3 (5)	1 (4)	2 (7)	0.352
